# Correlating Synthesis Parameters to Morphological Entities: Predictive Modeling of Biopolymer Aerogels

**DOI:** 10.3390/ma11091670

**Published:** 2018-09-09

**Authors:** Ameya Rege, Imke Preibisch, Maria Schestakow, Kathirvel Ganesan, Pavel Gurikov, Barbara Milow, Irina Smirnova, Mikhail Itskov

**Affiliations:** 1 Department of Continuum Mechanics, RWTH Aachen University, Kackertstraße 9, 52072 Aachen, Germany; itskov@km.rwth-aachen.de; 2 Institute of Thermal Separation Processes, Hamburg University of Technology, Eißendorfer Straße 38, 21073 Hamburg, Germany; imke.preibisch@tuhh.de (I.P.); pavel.gurikov@tuhh.de (P.G.); irina.smirnova@tuhh.de (I.S.); 3 Institute of Materials Research, German Aerospace Center, Linder Höhe, 51147 Cologne, Germany; Maria.Schestakow@dlr.de (M.S.); K.Ganesan@dlr.de (K.G.); Barbara.Milow@dlr.de (B.M.)

**Keywords:** aerogel, polysaccharide, pectin, cellulose, k-carrageenan, micromechanical, predictive model

## Abstract

In the past decade, biopolymer aerogels have gained significant research attention due to their typical properties, such as low density and thermal insulation, which are reinforced with excellent biocompatibility, biodegradability, and ease of functionalization. Mechanical properties of these aerogels play an important role in several applications and should be evaluated based on synthesis parameters. To this end, preparation and characterization of polysaccharide-based aerogels, such as pectin, cellulose and k-carrageenan, is first discussed. An interrelationship between their synthesis parameters and morphological entities is established. Such aerogels are usually characterized by a cellular morphology, and under compression undergo large deformations. Therefore, a nonlinear constitutive model is proposed based on large deflections in microcell walls of the aerogel network. Different sizes of the microcells within the network are identified via nitrogen desorption isotherms. Damage is initiated upon pore collapse, which is shown to result from the failure of the microcell wall fibrils. Finally, the model predictions are validated against experimental data of pectin, cellulose, and k-carrageenan aerogels. Given the micromechanical nature of the model, a clear correlation—qualitative and quantitative—between synthesis parameters and the model parameters is also substantiated. The proposed model is shown to be useful in tailoring the mechanical properties of biopolymer aerogels subject to changes in synthesis parameters.

## 1. Introduction

Biopolymer aerogels, specifically polysaccharide-based, have highly porous open cellular networks of naturally functionalized hydrocarbon materials. In the very first article on aerogels, in 1931, Kistler [1,2] described the preparation of such aerogels based on polysaccharides such as agar, nitrocellulose and cellulose. Since then, various polysaccharides such as cellulose, alginate, pectin, chitin, chitosan, carrageenan, agar, and starch have been exploited to prepare aerogels. The resulting aerogels have very high porosities, about 90–99% [3], and surface areas up to 600 m${}^{2}$·g${}^{-1}$ [4]. In this paper, amidated pectin, cellulose and k-carrageenan aerogels are discussed.

Pectin is a highly polar polysaccharide mainly consisting of galacturonic acid units linked via α –1–4 glucosidic binding. It can be extracted from apple and citrus fruits. Different types of pectin are widely used in food industry as thickening or gelling agents [5]. In the case of amidated pectin, parts of carbonic acid groups are substituted by methyl ester and amid groups. The gelation of amidated pectin can be performed by ionotropic gelation induced by polyvalent cations such as calcium ions [5]. Highly porous aerogels with high specific surface areas can thus be obtained.

Cellulose is an amphiphilic polysaccharide consisting of 1–4 linked β-d-glucopyranose with strong interaction, both inter and intramolecular via hydrogen bonds. It is predominantly produced by plants which makes it the most accessible biopolymer. Since cellulose is not soluble in common solvents, more complex solvents such as ionic liquids or molten salt hydrates are needed. The gelation of cellulose in aqueous zinc chloride solutions occurs upon leaving the distinct region of cellulose solubility which is accomplished by a decrease in temperature and by addition of water. After salt removal and followed by supercritical drying with CO_2_ pure cellulose aerogels can be obtained.

K-carrageenan is a linear sulfated polysaccharide having anhydrogalactopyranose ring in its monomer unit. Under controlled temperature, it undergoes reversible structural transition between helix to coil and vice versa. In its coil structure, the specific binding property of sulfate functional groups to the specific cations (such as potassium, rubidium or cesium) induces the formation of helix structures which results in a hard gel. Here the specific cations and their co-ions, i.e., anions can both act as potent helix stabilizers. Still the binding sites of cations and anions are not clearly reported. The Nuclear Magnetic Resonance (NMR) spectroscopy analyses and Boltzmann modeling studies suggested that the specific ions bind to the different class of sites on the helix [6,7,8,9]. Gelation temperature depends on the concentration of these specific cations and anions. The aerogels of k-carrageenan must be prepared with care perceiving the significance of interactions between specific ions, sulfate functional group and solvent molecules to avoid severe deformation and huge volume shrinkage from its original wet gel [4,10,11,12,13,14,15].

The properties of these polysaccharide-based aerogels make them very attractive to a wide spectrum of potential applications [16]. Their most cited application in literature is as carriers for drug delivery [3,17,18]. Recent studies show the suitability of polysaccharide-based aerogels for applications in advanced food materials, additives, and processing agents. Possible applications in food matrices are as carrier and encapsulation materials for sensitive components such as vitamins, flavors, or even bad tasting components to protect them against destruction during processing and storage. Due to high specific surface areas and high porosities, encapsulation of these components in aerogels is possible. Furthermore, controlled release in the body can be obtained due to given structural properties [19]. Another application for aerogels might be as structural elements of food matrices. This application requires the maintenance of given product properties such as structure, taste, and sensory attributes [20,21,22]. Moreover, all food applications require food-grade materials and processes throughout the production. Many aerogels of polysaccharides can be produced by fulfilling this requirement. In such applications, mechanical properties of aerogels would affect the structure and stability of food matrices. Furthermore, for the application as food packaging, mechanical properties, sorption capacity (hydrophilic or hydrophobic character) and thermal insulation are very important [19]. The porous system of polysaccharide-based aerogels also allows to use it as substrate or template materials in technical applications [23,24,25,26]. Cellulose aerogels are suitable templates or substrate materials to deposit either oxides via atomic layer deposition [23,24], metals via e.g., electroless plating [25] or polymers via addition of diluted polymers with the aim of reinforcement [26]. In a final step, the fibrillar network can either be removed from a given, e. g., oxide layer yielding nanotubular structures or be used, e. g., for microstructure exploration. Me-decorated cellulose aerogels can be used as catalytically active species which can be removed from the reaction media easily, simply extracting the monolith. Another interesting applicational field can be found in packed bed filters using polysaccharide-based aerogel beads.

Driven by the emerging fields of applications, particularly where the polysaccharide-based aerogels need to maintain their structural integrity or carry mechanical loads, there is a need for appropriate predictive modeling. So far only a few studies have investigated the mechanical behavior of polysaccharide-based aerogels. Typically, these aerogels undergo only small deformations followed by brittle failure under tension. A micromechanically motivated constitutive model describing the tensile response of cellulose aerogels was recently proposed in Rege and Itskov [27]. This model not only captures the mechanical behavior of cellulose aerogels, but also accurately predicts the onset of material failure. There, the network strain energy consists of both the bending and tension contributions. Of course, the model is limited to small deformations, which is sufficient for tensile loading (generally strains < 5%). Under compression, these aerogels can undergo large deformations ending in densification. A micromechanically motivated model was proposed in Rege et al. [28], to describe the compressive response of cellulose aerogels. This model is based on the bending of the cell wall fibrils, which was captured numerically using the Euler-Bernoulli beam theory for large deflections. Despite the simple and effective nature of the model, it has a few limitations, as for example, it does not account for the deformation due to the axial component of the load applied to the microcell. Literature on porous materials often postulates that the macroscopic behavior of porous materials is dictated by the microscopic bending of their cell walls. However, the axial effects can play an important role in the mechanical response of aerogels. In this paper, a nonlinear model is proposed, which accounts for the bending as well as the axial contribution of the load on the cell walls.

The paper is organized as follows. Section 2 deals with synthesis procedures of polysaccharide-based aerogels, their characterization and mechanical testing methods. Section 3 establishes correlations between the polysaccharide concentration and discusses its effect on the overall properties of these aerogels. A micromechanically motivated constitutive model is proposed and elucidated in Section 4. This is followed by a parameter study and model results discussion in the last sections.

## 2. Materials and Methods

### 2.1. Materials and Aerogel Preparation

#### 2.1.1. Pectin Aerogels

Materials used for the production of pectin aerogels are: amidated pectin (24% degree of esterification, 25% degree of amidation) kindly provided by Herbstreith & Fox KG, Neuenbürg, Germany. Sodium hydroxide and ethanol were purchased from Carl Roth GmbH & Co. KG, Karlsruhe, Germany. Light calcium carbonate was provided by Magnesia GmbH, Lüneberg, Germany. Carbon dioxide (99.9%) was purchased from AGA gas GmbH, Hamburg, Germany. Deionized water was used for dissolution of chemicals. All materials were used as received.

Aqueous amidated pectin solutions with 1, 2 and 3 wt. % amidated pectin (overnight magnetic stirring) were adjusted to pH 7 using 0.5 M sodium hydroxide solution. Calcium carbonate was added as cross-linker to a final concentration from 0.2 to 0.5 wt. % (referred to as cross-linking degree q = 1 for 1–3 wt. % pectin, respectively). Calcium carbonate particles were homogeneously suspended (Heidolph Diax 900, Schwabach, Germany) for approximately 2 min. Prepared pectin stock solutions were poured into polyethylene molds (1.7 cm height, 1.5, 2.2 and 4.0 cm diameter) and subjected to pressurized CO_2_ (5–6 MPa, room temperature) in a high-pressure autoclave. Pressure was maintained for about 24 h depending on the depth of pectin solution to ensure complete gelation (formation of hydrogel). Water in the hydrogels was replaced by ethanol in a stepwise solvent exchange. Successive ethanol/water baths (30, 60, 90, 100 wt. % EtOH) were used until a final ethanol concentration of minimum 98 wt. % inside the gel was reached (formation of alcogels). Ethanol from alcogels was extracted in a supercritical drying process with CO_2_. Alcogels were placed into a high-pressure autoclave and flushed with sc-CO_2_ at 55 ${}^{\circ }$C and 12 MPa and a flow rate of 20–80 g·min${}^{-1}$. Drying was performed for about 6 h to ensure complete removal of ethanol. Depressurization of the autoclave was performed with 1 bar·min${}^{-1}$.

#### 2.1.2. Cellulose Aerogels

All chemicals were used without further purification. Cellulose (degree of polymerization (DP) = 211, determined via cuprammonium hydroxide method) and zinc chloride (99 % water-free) were purchased from Sigma Aldrich, Taufkirchen, Germany. Deionized water was used for salt hydrate melt preparation (electrical conductivity below 0.5 μS·cm${}^{-1}$). Regeneration and washing, to remove the salt from the gel, was carried out in 2-propanol (Th. Geyer, 99%, technical grade) in volume ratios of 1:10 (gel:salt solvent), respectively. The salt removal was followed by electrical conductivity of the regeneration fluid. As soon as the spot test with silver nitrate was negative, the gels were subjected to supercritical drying extracting the solvent with sc-CO_2_ at 11.5 MPa and 45 ${}^{\circ }$C at a flow rate of 15 kg·h${}^{-1}$ for 7 h. Cellulose aerogels were prepared in 3, 5 and 7 wt. % cellulose content. A detailed description of cellulose aerogel preparation can be found in Schestakow et al. [29].

#### 2.1.3. K-Carrageenan Aerogels

K-carrageenan aerogels were prepared by following the procedure reported in Ganesan and Ratke [13]. All chemicals were used as received. Potassium thiocyanate and k-carrageenan were purchased from Merck and Sigma-Aldrich, respectively. For the preparation of hydrogels, deionized water was used. Acetone with a grade of Normapur was used for solvent exchange. Supercritical drying was carried out in an autoclave using pure CO_2_, following the procedure reported by Hoepfner et al. [30].

### 2.2. Characterization Methods

**Figure 1:**
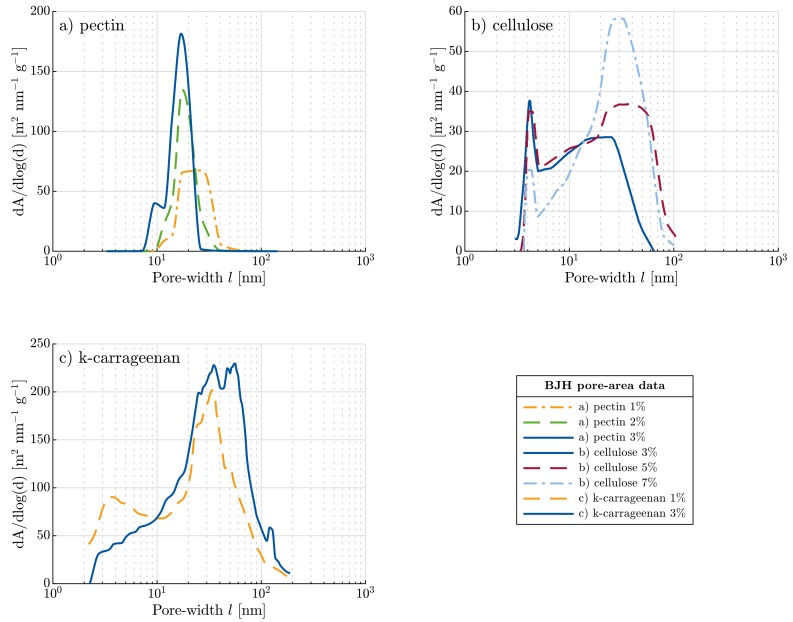
Pore size area distributions with varying biopolymer concentrations for polysaccharide-based aerogels: (**a**) pectin, (**b**) cellulose and (**c**) k-carrageenan.

**Figure A1:**
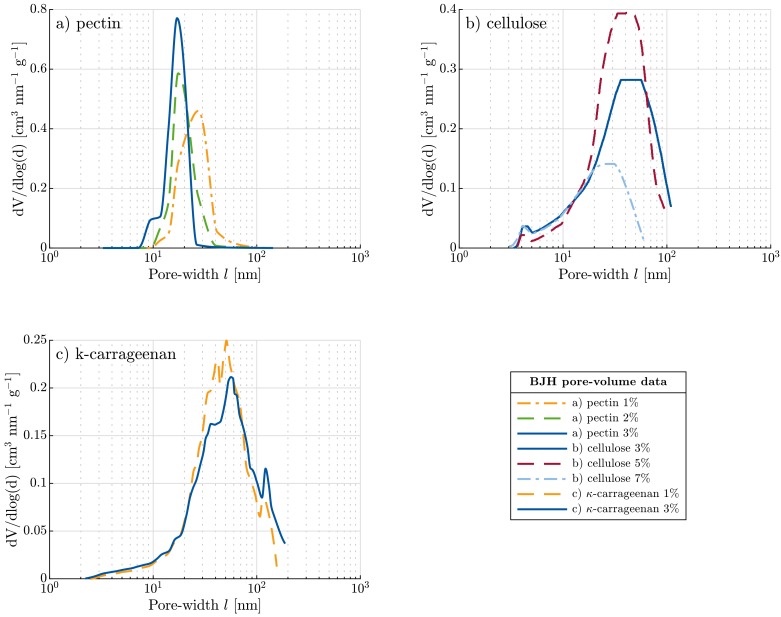
Pore size volume distributions with varying concentrations for three polysaccharide based aerogels: (**a**) pectin, (**b**) cellulose and (**c**) k-carrageenan.

All prepared aerogels were characterized with respect to their envelope (weight and volume) and skeletal densities, specific surface area, pore size distribution and average pore size. Microstructures were analyzed via scanning electron microscopy (SEM) after sputtering samples with a thin layer of gold. The preparation of cellulose aerogels for SEM analysis is crucial. As reported in [29], the nanofibers that make up the aerogel tend to form spindles and agglutination upon cutting. To assure brittle fracture and prevent these effects of cellulose nanofibers the samples were first placed in liquid nitrogen until the evolution of bubbles ceased. Then the samples were broken while still placed in the liquid nitrogen to preserve the apparent structure. Low-temperature nitrogen adsorption/desorption isotherms were measured to obtain specific surface area, pore size distribution and average pore size of aerogels via nitrogen physisorption using Brunnauer-Emmet-Teller (BET) measurements and Barrett-Joyner-Halends (BJH) calculations, respectively. Figure 1 shows the pore size area distributions (see also Figure A1 in Appendix B for the pore size volume distributions). They will be used later as input parameters to the proposed constitutive model. Envelope density of aerogel monoliths was determined from their weight and volume, while their skeletal density was measured via helium pycnometry. Aerogel porosity (ϵ) was calculated from envelope ($\rho_{e}$) and skeletal density ($\rho_{s}$) as follows:

(1) $$\epsilon =1-\frac{\rho_{e}}{\rho_{s}}$$

The obtained specific surface area ($S_{m}$) and $\rho_{s}$ are used to further determine the apparent average fibril diameter $d_{F}$ of the cell walls of all biopolymer aerogels in consideration, as follows [13,25,28],

(2) $$d_{F}=\frac{4}{\rho_{s}\cdot S_{m}}$$

and their calculated values, along with other quantities, are listed in Table 1.

### 2.3. Mechanical Testing

The mechanical response of all three polysaccharide-based aerogels was studied under uniaxial quasistatic compression. A compression rate of 1 mm·min${}^{-1}$ was applied for all aerogel specimens, which were of a cylindrical shape with 10 mm diameter and 10 mm height. Compression of pectin and cellulose aerogel monoliths was carried out on a universal testing machine Z010 by Zwick/Roell, Ulm, Germany, using a 1 kN load cell. Compression tests on k-carrageenan aerogel monoliths were performed on a universal testing machine Z03 from Latzke, Wiehl, Germany. For 1 and 3 wt. % of k-carrageenan aerogel samples, force heads with 100 N and 500 N, respectively, were used.

## 3. Concentration of Constituents and Its Effect on Aerogel Properties

### 3.1. Pectin Aerogels

The pectin concentration inside the stock solution influences aerogel properties (compare Table 1). In particular, envelope density increases from 0.04 to 0.09 g·cm${}^{-3}$ with increasing pectin concentration from 1 to 3 wt. % in stock solution, respectively. Due to increasing envelope density, porosity and specific pore volume of aerogel samples decrease. Calculated porosities are 97%, 96% and 93% for 1, 2 and 3 wt. % pectin in stock solutions, respectively. Measured specific volumes of mesopores are 9.7, 5.9 and 4.4 cm${}^{3}$·g${}^{-1}$, respectively.

The skeletal density decreases slightly with increasing pectin concentration in stock solution. Nevertheless, it is almost independent of the pectin concentration. These results suggest that the skeletal density results from gel formation mechanism and pectin chain interaction rather than from the pectin concentration.

**Figure 2:**
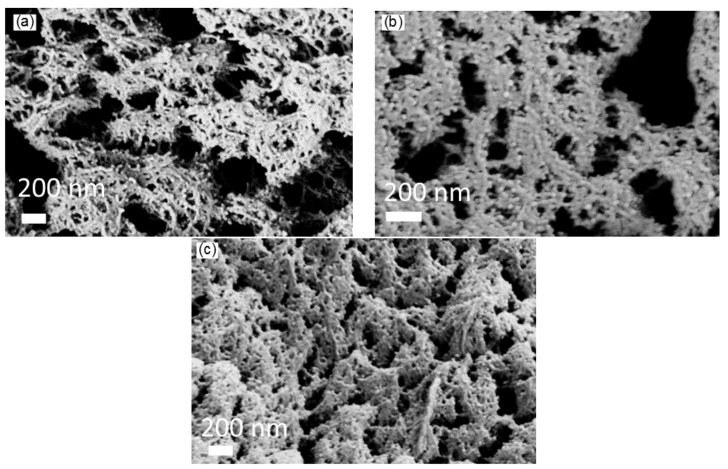
SEM images of amidated pectin aerogels with (**a**) 1 wt. %, (**b**) 2 wt. %, (**c**) 3 wt. % pectin concentration.

This observation is confirmed also by the measurement of fibril diameter which is only slightly dependent on the pectin concentration. Both the results suggest that the solid structure of pectin aerogels is formed from chain interactions and the gelation mechanism rather than from the agglomeration of pectin chains due to the increased pectin concentration. Higher pectin concentrations instead lead to more cross-links inside the aerogel structure which can be observed from the change of average pore size of aerogels with different concentrations. For low pectin concentration, average pore size is higher (see Figure 1) and pore size distribution is narrow, compared to cellulose and k-carrageenan aerogels. Increasing pectin concentration (with constant $\rho_{s}$ and $d_{F}$) causes an increase of the number of fibrils and, therefore, decrease of the pore diameter. Specific surface area of pectin aerogels increases slightly with decreasing pectin concentration; however, it is similar for 1 and 2 wt. % pectin in stock solution (around 710 m${}^{2}$·g${}^{-1}$). The slight decrease at higher pectin concentration (627 m${}^{2}$·g${}^{-1}$) might be explained by less pores and, therefore, lower porosity, due to higher solid content (see also Figure 2).

Strong volume shrinkage of cylindrical pectin hydrogels occurs throughout solvent exchange and supercritical drying. Aerogel volume compared to hydrogel volume was evaluated and 79.8 ± 0.6%, 72.5 ± 2.6% and 71.6 ± 0.4% of volume shrinkage were measured for 1,2 and 3 wt. % pectin, respectively. It can be observed that the increase of the pectin concentration results in a decrease of the volume shrinkage. This might be explained by the increased number of interactions between pectin chains at higher pectin concentrations and higher stability of these gels. Furthermore, it should be noted that the drying method has a drastic influence on the morphological parameters of all aerogels considered, and thus influences their mechanical properties [31].

### 3.2. Cellulose Aerogels

The dissolution of cellulose in an appropriate media strongly depends on the DP of the cellulose used. Zinc chloride salt hydrate melts are suitable solvents for cellulose, even for high DP cellulose such as bacterial cellulose. In the present study, we focus on cellulose aerogels consisting of 3, 5 and 7 wt. % solid content.

The increasing solid content from 3 to 7 wt. % is accompanied by an almost linear increase in envelope density of the resulting cellulose aerogels, from 0.16 to 0.28 g·cm${}^{-3}$ respectively. Meanwhile, the skeletal density is not measurably affected showing values of 1.49 g·cm${}^{-3}$ independent of the solid content.

According to the observations in other polysaccharide-based aerogels, the porosity of cellulose aerogels decreases from 90 to 82% with rising solid content since the pore volume reduces from 2.9 to 2.2 cm${}^{3}$·g${}^{-1}$. This is due to the increase in number of fibrils per unit cell, which cannot however entirely explain the rather minor decrease in pore volume of cellulose aerogels. In this case, the fibril diameter is another factor that leads to a comparatively small shift in the pore volume. This is attributed to the increasing shell surface of the nanofibrils resulting from the increasing diameter which becomes apparent from both, SEM analysis and calculations from BET results. Both methods confirm the noticeable increase in fibril diameter from 5.5 nm with 3 wt. % cellulose aerogels to 7.6 nm with 7 wt. %.

As a result of the increase in fibril diameter, the volume of the solid content increases, subsequently reducing the specific surface area and affecting the pore size distribution. BJH data shown in Figure 1b) clearly demonstrate that the pore size area distribution of cellulose aerogels is predominantly bimodal, with a narrow peak around 4 nm and broad peaks between 20 and 35 nm. Cellulose aerogels of 3 wt. % show a dominating peak at 4 nm while 7 wt. % have a dominant peak at 30 nm. In contrast, 5 wt. % cellulose aerogels depict a good balance of these two pore sizes. The ratio of these peaks appears to be a function of the solid content. The lower the cellulose content is, the higher the peak of the small pores becomes, while an increase in solid content results in rather coarse pores.

**Figure 3:**
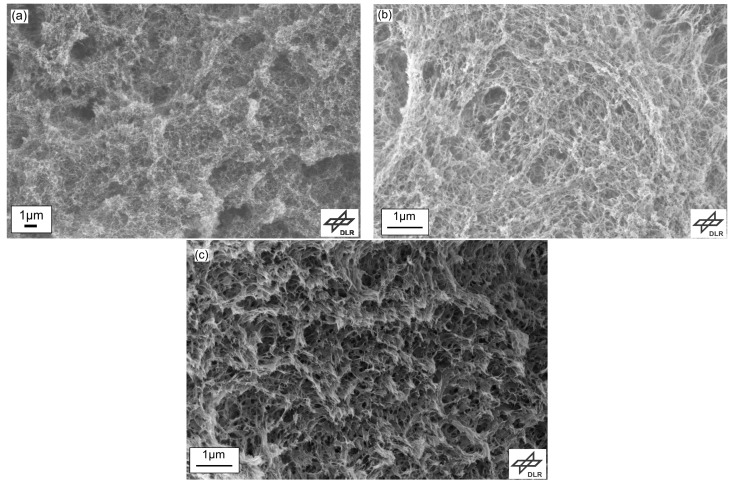
SEM images of cellulose aerogels with (**a**) 3 wt. %, (**b**) 5 wt. %, (**c**) 7 wt. % cellulose concentration.

This pore size distribution suggests that fibrils of larger diameters may trigger some of the small pores to collapse and move together and thus agglomerate, which leads to the coarsening of the pore size. These results indicate a different mechanism of the gel formation in cellulose aerogels from zinc chloride than observed in e.g., pectin aerogels making it a subject matter of current research. Nevertheless, the structures observed in SEM, see Figure 3, are very well comparable due to the fibrillar nanofelt-like nature of polysaccharide aerogels.

### 3.3. K-Carrageenan Aerogels

**Figure 4:**
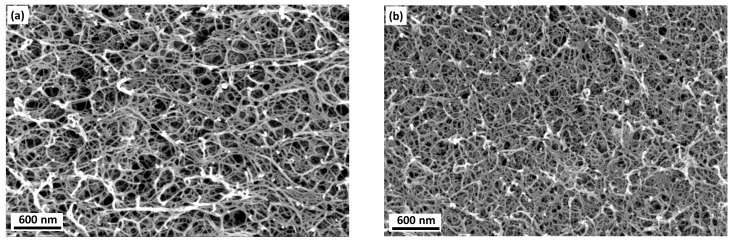
SEM images of k-carrageenan aerogels: (**a**) 1 wt. %, (**b**) 3 wt. % k-carrageenan concentration.

K-carrageenan aerogels were prepared with 1 and 3 wt. % according to the procedure reported in Ganesan and Ratke [13]. The physical properties of k-carrageenan aerogels are summarized in Table 1. The washing and the drying processes induce the volume shrinkage of samples which was measured from the wet hydrogel cylindrical body to the dry aerogel body. The total volume shrinkage was observed to be about 68.2% and 63.8% for 1 and 3 wt. %, respectively. By increasing the concentration from 1 to 3 wt. %, the degree of volume shrinkage reduces only by about 4%. In comparison with other non-ionic polysaccharide aerogels such as cellulose, k-carrageenan aerogels showed relatively large volume shrinkage which is due to the presence of anionic sulfate functional groups. SEM images (Figure 4) show the microstructure of k-carrageenan aerogels. The pore size was in the range from meso- to macropores. The aerogels exhibited nano-felt interconnected fibrillar structure similar to other polysaccharide aerogels. In comparison with 1 wt. % of k-carrageenan aerogels, 3 wt. % of k-carrageenan showed tightly bound nanofibrillar network, because of the volume shrinkage.

The porous structure and the BET specific surface area were analyzed by nitrogen adsorption-desorption isotherms (Table 1). Type IV isotherm was found similar to the data discussed in the literature [13]. The average specific surface area was observed to be 222 m${}^{2}$·g${}^{-1}$. The average fibril diameter was estimated to be about 10.5 nm which was calculated using skeletal density of k-carrageenan according to (2). The pore size area distributions of 1 and 3 wt. % of k-carrageenan aerogels are shown in Figure 1c. A broad pore size distribution is observed there. As BJH pore volume approximation is valid only for mesopores (2–50 nm), the data showed major pore size distribution peaks beyond 50 nm and a peak or shoulder in the range of 25–45 nm, which is in good agreement with the results of SEM. For 1 wt. % of k-carrageenan aerogel, the envelope density was about 0.08 g·cm${}^{-3}$ whereas it was 0.15 g·cm${}^{-3}$ for 3 wt. % of k-carrageenan aerogel. The density increased with the solid fraction. Meanwhile the porosity decreases from 96% to 91% when the concentration of k-carrageenan was varied from 1 to 3 wt. %.

## 4. Micromechanical Constitutive Model

A generalized constitutive model describing the compressive response of polysaccharide-based aerogels is proposed in this section based on a microcell approach previously used in Rege et al. [28] for cellulose aerogels under compression. In the previous modeling approach, only the orthogonal component of the applied force (responsible for bending of the cell wall fibril) was considered as a first approximation. This means that the tension/compression component responsible for the axial compression of the cell wall fibril was first disregarded. The bending behavior of cell wall fibrils was captured using the extended version of the Euler-Bernoulli beam theory for large deflections [32]. A numerical approximation [33] to the analytical solution was implemented and a bending energy-based model was proposed. In the presented model, we additionally take the tension/compression contribution into account and use an analytical approach to describe the nonlinear behavior of cell wall fibrils.

### 4.1. Nonlinear Behavior of a Microcell under Compression

**Figure 5:**
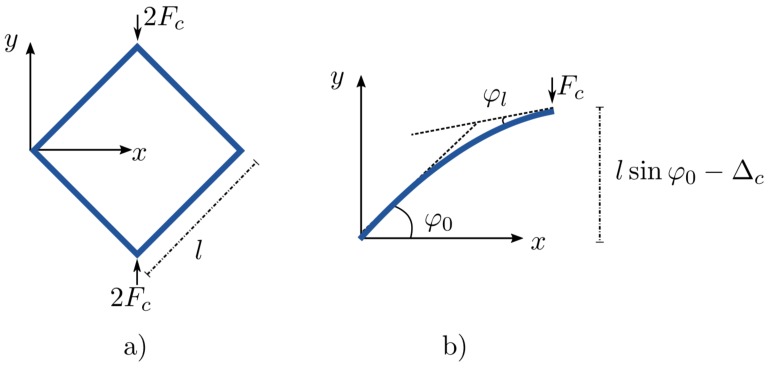
(**a**) Square shaped microcell subject to combined bending and compression, (**b**) free body diagram of a single microcell wall (beam) subject to large deflection.

We consider a square shaped microcell illustrated in Figure 5a. The solution to the deflections of such square shaped frames undergoing large deformations was first expressed by Jenkins et al. [34]. This solution is analytical and expressed using elliptic integrals (see Appendix A), which were then solved in MAPLE (Maple 2017. Maplesoft, a division of Waterloo Maple Inc., Waterloo, ON, Canada). Furthermore, a simplified relation between the normalized force and the normalized displacement is proposed via a polynomial fit and can be expressed as:

(3) $$\frac{F_{c}l^{2}}{EI}=\left(1.086{\left(\frac{\Delta_{c}}{l}\right)}^{3}-2.1236{\left(\frac{\Delta_{c}}{l}\right)}^{2}+2.8104\left(\frac{\Delta_{c}}{l}\right)\right)$$

where $F_{c}$ and $\Delta_{c}$ are the applied force and resulting deflection (see Figure 5) respectively, *E* and *I* are Young’s modulus and the second moment of inertia of the fibril respectively, while *l* is the length of the fibril.

**Remark** **1.** The above proposed formula (Equation 3) is a polynomial fit to the analytical solution and is verified up to a normalized displacement of $\frac{\Delta_{c}}{l}=1$, which is already very large deflection. Also, the obtained coefficients of polynomial terms are valid only for the case of $\varphi_{0}=45^{\circ }$.

**Figure 6:**
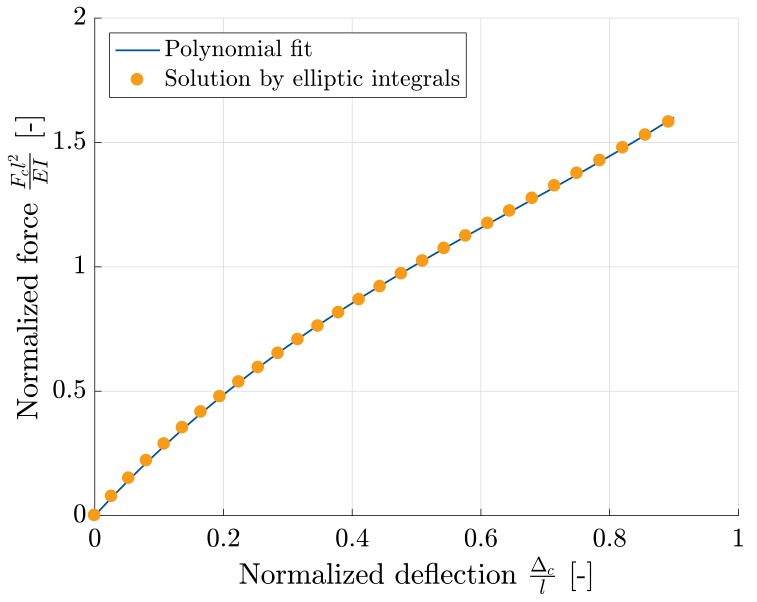
Comparison of the analytical solution for nonlinear beam under combined loading against the proposed polynomial fit.

This expression is plotted against the elliptic solution from MAPLE in Figure 6. The strain energy ψ of such a fibril in Figure 5b is equal to the amount of work done by the force applied to it. Accordingly,

(4) $$\begin{array}{cc} \psi & =\int_{0}^{\Delta_{c}}F_{c}d\Delta^{*} \end{array}$$

(5) $$\begin{array}{c} =\frac{EI}{l^{3}}\left(0.27\left(\frac{\Delta_{c}^{4}}{l^{2}}\right)-0.71\left(\frac{\Delta_{c}^{3}}{l}\right)+1.41\left(\Delta_{c}^{2}\right)\right) \end{array}$$

The deflection of the microcell can further be expressed in terms of the applied microstretch λ. Accordingly, the strain energy function for a whole microcell can be represented as a function of λ and *l* as:

(6) $$\psi \left(\lambda ,l\right)=\frac{2EI}{l}\left(a_{1}{\left(1-\lambda \right)}^{4}-a_{2}{\left(1-\lambda \right)}^{3}+a_{3}{\left(1-\lambda \right)}^{2}\right)$$

where $a_{1}=0.135,\;a_{2}=0.5,\;a_{3}=1.41$ are geometrical (material independent) constants for the case of $\varphi_{0}=45^{\circ }$. Note that the above defined strain energy now combines the bending strain energy and the tension/compression strain energies.

### 4.2. Network Portrayal and Description of Pore Collapse

**Figure 7:**
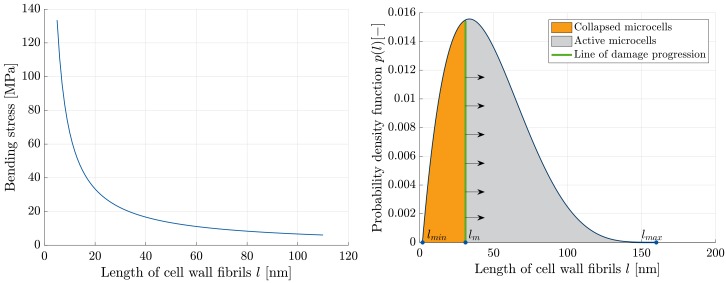
**Left**: Maximal bending stress versus the cell wall fibril size, for example, for λ=0.95. **Right**: An illustration of the cell-size distribution. The area under the curve encompasses the microcells in the network. Accordingly, with progressive damage, the number of collapsed microcells increases.

The aerogel network is assumed to be made up of such idealized square shaped microcells of varying cell wall sizes. The distribution of microcells throughout the network is considered to be homogeneous and isotropic. The varying cell sizes are obtained from nitrogen desorption isotherms, as described in Section 2 and then are approximated by a generalized beta probability distribution function by

(7) $$p\left(l\right)=\frac{{\left(l-l_{min}\right)}^{\alpha -1}{\left(l_{max}-l\right)}^{\beta -1}}{B\left(\alpha ,\beta \right){\left(l_{max}-l_{min}\right)}^{\alpha +\beta -1}}$$

where α and β are shape parameters that can be obtained by fitting to the BJH data [28]. Damage of the aerogel network is explained by pore collapse as follows. A cell wall fibril is assumed to break once the bending stress reaches a critical value, which results in microcell (pore) collapse. This bending stress is plotted against the cell wall sizes in Figure 7 (left). Accordingly, at the same applied stretch, smaller microcells would collapse first. Due to the collapse of smaller microcells, the probability density function of *l* evolves with the deformation as illustrated in Figure 7 (right).

Now, the network strain energy in any spatial direction d can be given by

(8) $$\overset{d}{\Psi }=\int_{l_{m}}^{l_{max}}N_{0}p\left(l\right)\psi \left(l,\overset{d}{\lambda }\right)dl$$

where $N_{0}$ denotes the initial total number of microcells within the network and p(l) is given by Equation (7). $\overset{d}{\lambda }$ denotes the stretch in the direction d. The integration is carried out only over the active microcells lying within the interval $\left[l_{m},l_{max}\right]$. Note that in the reference (undeformed) state, $l_{m}=l_{min}$. At very high compressive strains, the collapsed microcells cause the densification of the aerogel network which is however not accounted for by the model.

The 1D network energy presented above can be generalized to a 3D one by numerical integration over the unit sphere [35]. Such numerical integration schemes can be considered as an accurate and reliable tool for full network models if the latter ones are based on a continuous directional distribution of elements (microcells in this case), and their response is not highly nonlinear [36]. Accordingly, the total network strain energy can be formulated as

(9) $$\Psi \approx \sum_{i=1}^{g}\omega_{i}\int_{l_{m}}^{l_{max}}N_{0}p\left(l\right)\psi \left(l,\overset{d_{i}}{\lambda }\right)dl$$

where $\omega_{i}$ specifies the weighting factors corresponding to the integration directions $d_{i}\;\left(i=1,2,\ldots g\right)$. A numerical algorithm by Heo and Xu [37] with g=45 directions is used. Now, the macroscopic nominal stress can be expressed in terms of the first Piola-Kirchhoff (1st PK) stress tensor P as:

(10) $$P=\frac{\partial \Psi }{\partial F}=\frac{\partial \Psi }{\partial \overset{d_{i}}{\lambda }}\frac{\partial \overset{d_{i}}{\lambda }}{\partial F}$$

where F represents the macroscopic deformation gradient. The solution to the term $\frac{\partial \overset{d_{i}}{\lambda }}{\partial F}$ is given by $\frac{\partial \overset{d_{i}}{\lambda }}{\partial F}=F\;d_{i}⊗d_{i}$ (see, e.g., [27]).

## 5. Parameter Study

The presented model includes six micromechanically motivated parameters as listed in Table 2. The first four parameters $l_{min}$, $l_{max}$, $d_{F}$ and *E* are all material constants. Of these, the first two are obtained from the limits of the BJH pore size area distribution data. Besides image evaluation, the diameter $d_{F}$ is obtained as discussed in Section 2. Finally, *E* is evaluated using the Gibson-Ashby approach of subtracting the pore volume from the total material.

$N_{0}$ and $\sigma_{max}$ are fitting parameters and separated in Table 2 by a line. The total number of microcells in the network depends on the specimen size. Such size effects/size sensitivity are common to porous cellular materials. The critical bending stress is a material parameter and could be estimated by means of MD simulations. For predictive modeling, it is important that every material parameter can be tailored during the synthesis procedure. In our modeling approach, this is the case with four out of the six material parameters (listed above in Table 2). Given successful quantitative interpretation of the last two parameters, the model shows the capability to predict the constitutive behavior of any polysaccharide-based aerogel.

**Figure 8:**
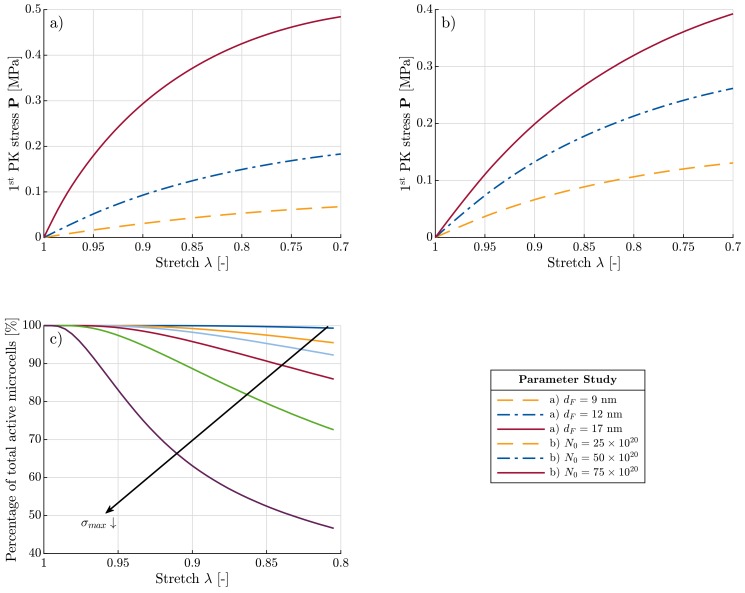
Sensitivity analysis of material parameters: (**a**) and (**b**) stress-stretch diagrams for different values of $d_{F}$ and $N_{0}$, respectively, (**c**) percentage of the active microcells versus stretch for different allowable bending stress values.

Furthermore, it is important to understand the sensitivity of the network response to changes in the model parameters. $d_{F}$ and $N_{0}$ have a direct relation to synthesis parameters of the polysaccharide-based aerogel. $N_{0}$ only has a multiplicative effect and does not change the form of the stress-stretch curve (see Figure 8b). On the contrary, $d_{F}$, while showing similar stiffening upon increase, also influences the form of the curve (see Figure 8a). Due to increasing $d_{F}$, the critical bending stress of the microcell walls, is reached sooner. This results in rapid pore collapse, which accordingly expedites the network softening. Thus, $d_{F}$ plays an important role not only in the energy function but also in the damage of the aerogel network.

Another critical parameter in the model is $\sigma_{max}$. This has the largest influence on controlling the collapse of the microcells, and thus the network (see Figure 8c). Unfortunately, no correlation between this factor and the synthesis parameters has yet been established. Identification of such parameters will enable to control the network behavior subject to altering syntheses.

## 6. Model Predictions vs. Experimental Data

As mentioned in Section 2 and Section 3, pectin aerogels with concentrations—1, 2 and 3 wt. %, cellulose aerogels with concentrations—3, 5 and 7 wt. %, and k-carrageenan aerogels with concentrations—1 and 3 wt. %, were tested under uniaxial quasistatic compression. No Poisson’s effect was observed in the case of all types of aerogels. This has already been demonstrated via optical measurements for cellulose aerogels [27]. Furthermore, Young’s modulus and yield stress increased with increasing relative density and decreasing porosity. For example, in case of k-carrageenan aerogels, as the concentration increased from 1 to 3 wt. %, the relative density and Young’s modulus increased by a factor 2.2 and 3.6, respectively. This shows a direct correlation between the synthesis parameters on the one hand and the structural and mechanical properties on the other hand.

**Figure 9:**
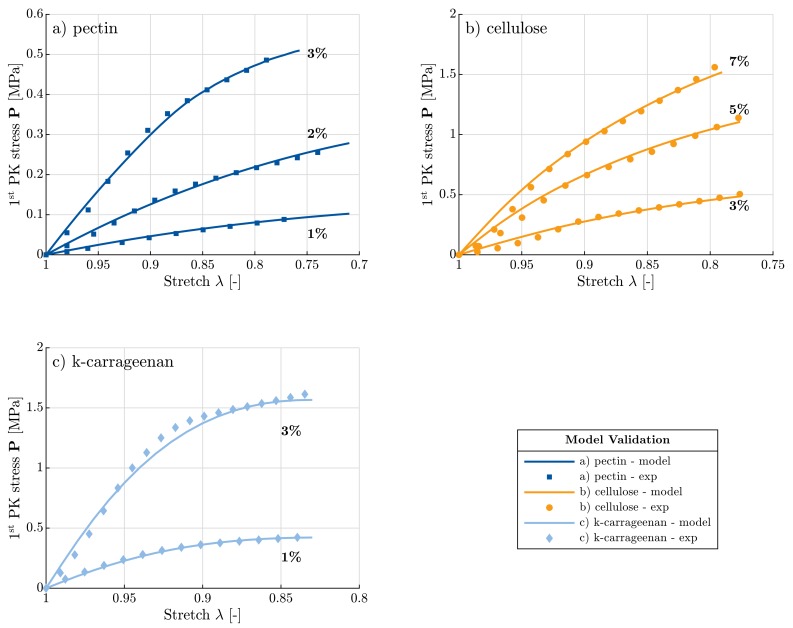
Model predictions versus experimental data for three polysaccharide-based aerogels: (**a**) pectin, (**b**) cellulose and (**c**) k-carrageenan.

Furthermore, the model results have been validated against the obtained experimental data. In Figure 9, predictions of the model are plotted against experimental data of the three different types of polysaccharide-based aerogels, each with different concentrations. Good agreement with the experimental results can be observed for all aerogels and for every concentration. The agreement is even better in comparison to the previous model [28] (see Figure S1 in the Supplementary Material for direct comparison of both model predictions against experimental data of cellulose aerogels prepared from calcium-thiocyanate route). This development is a result of the improved model, which now takes into account not only the bending energy, but also the tension/compression energy. A comparison of Young’s moduli values, obtained from the experimental and predicted stress-stretch curves, are listed in Table 3 for the different biopolymer aerogels. The validation between the two values gives a measure of the quality of fit of our model to the experimental data. Although several pectin and pectin-based hybrid aerogels have been characterized in terms of Young’s modulus [38,39,40,41], a direct comparison is difficult due to different drying methods (freeze drying) and the presence of a second component. To the best of our knowledge, only one study by Rudaz et al. [41] reports data for pure pectin aerogels dried supercritically. Young’s moduli for pectin reported in Table 3 are very close to the values in [41] at corresponding densities. This provides an additional verification of our model results.

## 7. Conclusions

The paper presents an enhancement of constitutive modeling, not only for cellulose aerogels but in general to nano-fibrillary structured polysaccharide-based aerogels featuring pectin, cellulose, and k-carrageenan. First, the dependency of several properties of biopolymer-based aerogels, such as density and pore size distribution, on the biopolymer concentration has been established. All biopolymer-based aerogels show clearly increasing density and stability with increasing polysaccharide concentration. The specific surface area decreases for amidated pectin and cellulose aerogels, but slightly increases in the case of k-carrageenan aerogels. The apparent average fibril diameter shows slight increments with increasing polysaccharide concentration, while the network of microcells also gets denser. Both these factors result in a decreasing porosity with increasing biopolymer concentration. These dependencies of biopolymer-based aerogels have been successfully incorporated into the constitutive model for prediction of their mechanical behavior. The proposed model is based on square shaped microcells and construes the response of nanofibers within a complex network. Only a few parameters, such as fibril length, diameter, and the number of microcells, are needed to get a good agreement of the model predictions to the experimental data. In particular, four out of six model parameters can be qualitatively as well as quantitatively used directly to alter the synthesis procedure to obtain a desired response. The correlation of the remaining two constants to the synthesis parameters should further be studied. Nevertheless, the proposed model approach shows promising predictive capabilities and an improvement in making it more robust remains the primary outlook of this work.

## Figures and Tables

**Table 1:** Textural properties of polysaccharide-based aerogels.

	Conc.	$S_{m}$	$\rho_{e}$	$\rho_{s}$	$V_{p}$	$\epsilon {\;}^{1}$	$d_{F}$
	[wt. %]	[m${}^{2}$·g${}^{-1}$]	[g·cm${}^{-3}$]	[g·cm${}^{-3}$]	[cm${}^{3}$·g${}^{-1}$]	[%]	[nm]
	1	718 ± 30	0.04 ± 0.02	1.60 ± 0.04	9.7 ± 0.1	97 ± 3	3.4
Pectin ${}^{2}$	2	710 ± 30	0.07 ± 0.02	1.60 ± 0.04	5.9 ± 0.1	96 ± 3	3.5
	3	627 ± 30	0.09 ± 0.02	1.40 ± 0.04	4.4 ± 0.1	94 ± 3	4.0
	3	491 ± 25	0.16 ± 0.01	1.49 ± 0.01	2.9 ± 0.1	90 ± 2	5.5
Cellulose	5	421 ± 23	0.22 ± 0.02	1.49 ± 0.01	2.4 ± 0.1	86 ± 2	6.4
	7	354 ± 28	0.28 ± 0.02	1.49 ± 0.01	2.2 ± 0.1	82 ± 2	7.6
	1	218 ± 3	0.08 ± 0.01	1.72 ± 0.01	1.2 ± 0.1	96 ± 1	10.6
K-carrageenan	3	230 ± 9	0.15 ± 0.01	1.72 ± 0.01	1.3 ± 0.1	91 ± 1	10.1

^1^ For pectin, the values were calculated by propagation of error; ^2^ The standard deviations for BET specific surface, envelope and skeletal densities, and for specific pore volume represent a usual precision of measurements.

**Table 2:** Description of material parameters in the presented model.

Parameter	Description
$l_{min}$, $l_{max}$	Minimum and maximum cell wall fibril lengths
$d_{F}$	Average cell wall fibril diameter
*E*	Young modulus of the cell wall fibril
$N_{0}$	Initial number of microcells in the network
$\sigma_{max}$	Critical bending stress in the cell wall fibrils

**Table 3:** Interpretation of Young’s modulus from the experimental and model stress-stretch curves in Figure 9.

	Conc.	Young’s Modulus—Exp.	Young’s Modulus—Model
	[%]	[MPa]	[MPa]
	1	0.51 ± 0.05	0.51 ± 0.03
Pectin	2	1.35 ± 0.05	1.35 ± 0.06
	3	3.2 ± 0.2	3.2 ± 0.2
	3	2.7 ± 0.2	2.9 ± 0.2
Cellulose	5	7.5 ± 0.6	7.6 ± 0.6
	7	11.4 ± 0.9	11.2 ± 0.5
K-carrageenan	1	4.9 ± 0.6	4.6 ± 0.6
	3	17.6 ± 0.6	18 ± 1

## Supplementary Materials

The following are available online at http://www.mdpi.com/1996-1944/11/9/1670/s1, Figure S1: (**Left**): predictions of the previous bending based model vs. experimental data of CT based cellulose aerogel (reproduced from [1] with permission from the Royal Society of Chemistry). (**Right**): predictions of the newly proposed model (accounting for the bending as well as the compression contributions to the strain energy function) vs. the same experimental data of CT based cellulose aerogel.

## Abbreviations

The following abbreviations are used in this manuscript:

NMR	Nuclear Magnetic Resonance
DP	Degree of polymerization
BET	Brunnauer-Emmet-Teller measurements
BJH	Barrett-Joyner-Halenda
1D	One-dimension
3D	Three-dimensions
MD	Molecular dynamics
PK	Piola-Kirchhoff

## Appendix A. Solution to Large Deflection of the Square Shaped Microcell

The governing Equation for bending of a microcell wall fibril under compressive loading (refer to Figure 5) can be expressed by

(A1) $$\frac{d\varphi }{ds}=\frac{F_{c}}{EI}\left(l\cos\varphi_{0}+\Delta_{ch}-x\right)$$

where φ is the angle between the tangent to the deflected member at a point and the *x* axis, and $\Delta_{ch}$ is the horizontal displacement of the fibril subject to compression. Also, $\varphi_{0}$ is the angle at the rigid joint. A new variable θ is then defined as

(A2) $$\sin^{2}\theta =\frac{\left(1-\sin\varphi \right)}{2k^{2}}$$

where

(A3) $$2k^{2}=1-\sin\varphi_{l}$$

and $\phi_{l}$ is the angle at the joint where the force is applied. The following Equations are now obtained using elliptic integrals

(A4) $$\eta \left(\sin\varphi_{0}-\frac{\Delta_{c}}{l}\right)=K\left(k\right)-F\left(\theta_{1},k\right)-2E\left(k\right)+2E\left(\theta_{1},k\right)$$

(A5) $$\eta \left(\cos\varphi_{0}+\frac{\Delta_{ch}}{l}\right)=-2k\cos\theta_{1}$$

(A6) $$\eta =F\left(\theta_{1},k\right)-K\left(k\right)$$

(A7) $$\eta \frac{y}{l}=F\left(\theta ,k\right)-F\left(\theta_{1},k\right)-2E\left(\theta ,k\right)+2E\left(\theta_{1},k\right)$$

(A8) $$\eta \frac{x}{l}=2k\left(\cos\theta -\cos\theta_{1}\right)$$

where

(A9) $$\sin^{2}\theta_{1}=\frac{\left(1-\sin\varphi_{0}\right)}{1-\sin\varphi_{l}}$$

and

(A10) $$\eta^{2}=\frac{F_{c}l}{EI}$$

The above defined new elliptic Equations were solved in MAPLE and the resulting curve for the force-deflection (normalized) solution was fit to a polynomial (as described in Equation (3)) and is plotted in Figure 6.

## Appendix B. Pore size Volume Distribution

The proposed model uses pore size area distributions (see Figure 1) as input parameters. Additionally, pore-size volume distributions are plotted in Figure A1 for reference.
