# Supplementary material for: Correlating Synthesis Parameters to Morphological Entities: Predictive Modeling of Biopolymer Aerogels
Source: Materials (Basel). 2018 Sep 9;11(9):1670. doi: 10.3390/ma11091670 (PMC6163492; doi:10.3390/ma11091670)
Supplement: Supplementary file 1 [file materials-11-01670-s001.zip › SupplementaryMaterial/Supplementary Material/Supplementary_Material.pdf]

Supplementary Material – Correlating Synthesis Parameters to Morphological Entities: Predictive Modeling of Biopolymer Aerogels

Ameya Rege[1,*], Imke Preibisch[2], Maria Schestakow[3], Kathirvel Ganesan[3], Pavel Gurikov[2], Barbara Milow[3], Irina Smirnova[2] and Mikhail Itskov[1]

[1]Department of Continuum Mechanics, RWTH Aachen University, Kackertstraße 9, 52072 Aachen, Germany
[2]Institute of Thermal Separation Processes, Hamburg University of Technology, Eißendorfer Straße 38, 21073 Hamburg, Germany
[3]Institute of Materials Research, German Aerospace Center, Linder Höhe, 51147 Cologne, Germany

The predictions of the proposed constitutive model, versus the experimental data of calcium thiocyanate (CT) based cellulose aerogels, are compared with the predictions of the previous model, versus the same data, published in Rege et al. [1] (see Figure S1). It is seen that the curve from the model predictions now is in much better agreement, qualitatively as well as quantitatively, with the experimental data than the previous model. The effect of introducing the axial component to the strain energy function is hereby visible.

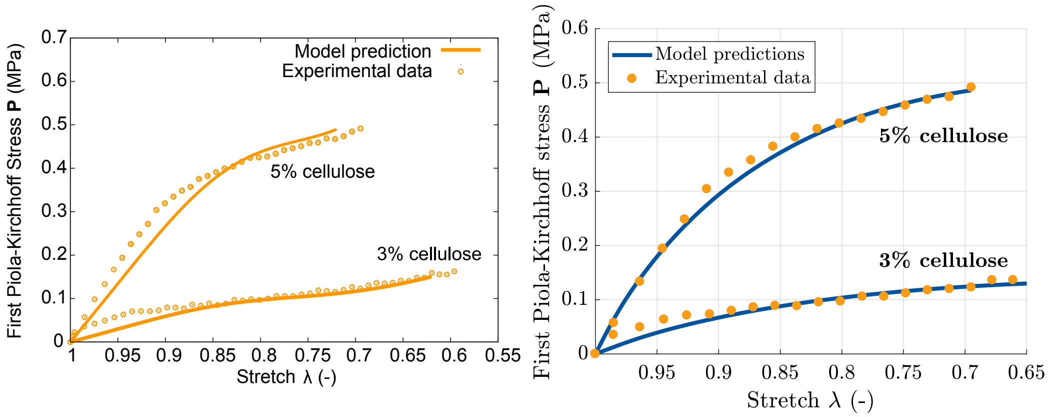

Figure S1. (**Left**): predictions of the previous bending based model vs. experimental data of CT based cellulose aerogel (reproduced from [1] with permission from the Royal Society of Chemistry). (**Right**): predictions of the newly proposed model (accounting for the bending as well as the compression contributions to the strain energy function) vs. the same experimental data of CT based cellulose aerogel

[1] Rege, A.; Schestakow, M.; Karadagli, I.; Ratke, L.; Itskov, M. Micro-mechanical modelling of cellulose aerogels from molten salt hydrates. *Soft Matter* **2016**, *12, 7079-7088.*
