# Supplementary figures and images for: Correlating Synthesis Parameters to Morphological Entities: Predictive Modeling of Biopolymer Aerogels
Source: Materials (Basel). 2018 Sep 9;11(9):1670. doi: 10.3390/ma11091670 (PMC6163492; doi:10.3390/ma11091670)

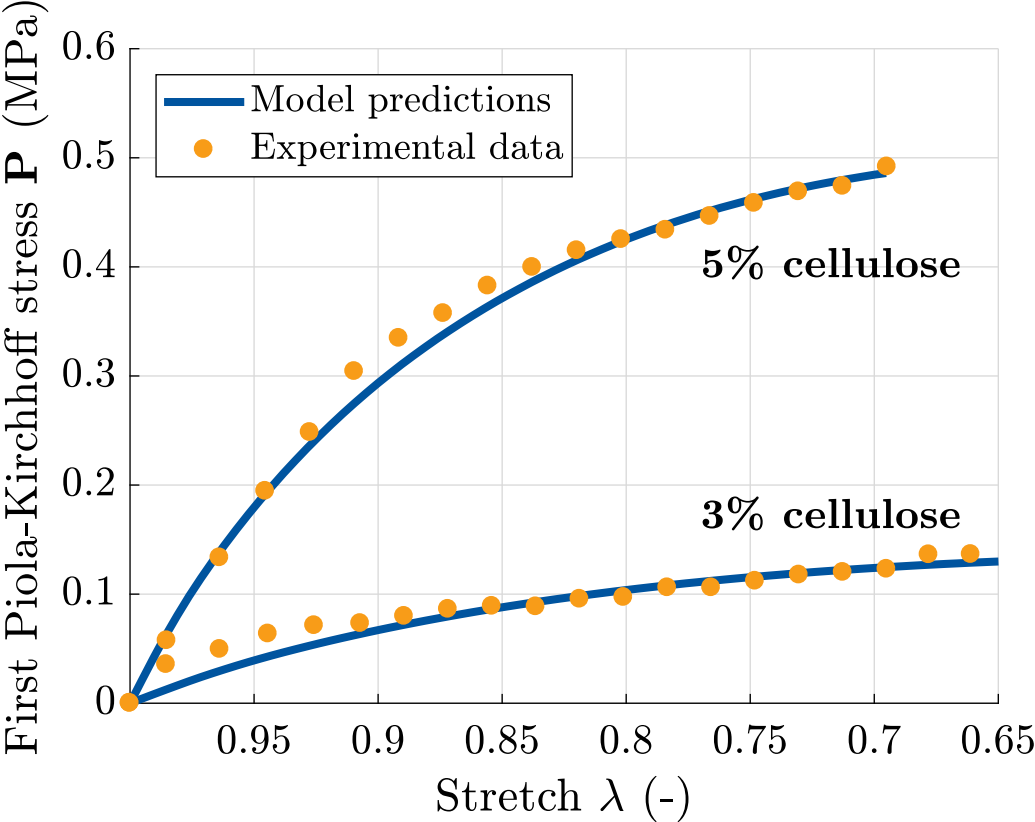

Supplement: Supplementary file 1 [file materials-11-01670-s001.zip › SupplementaryMaterial/Supplementary Material/celluloseCAModExp-eps-converted-to.pdf]

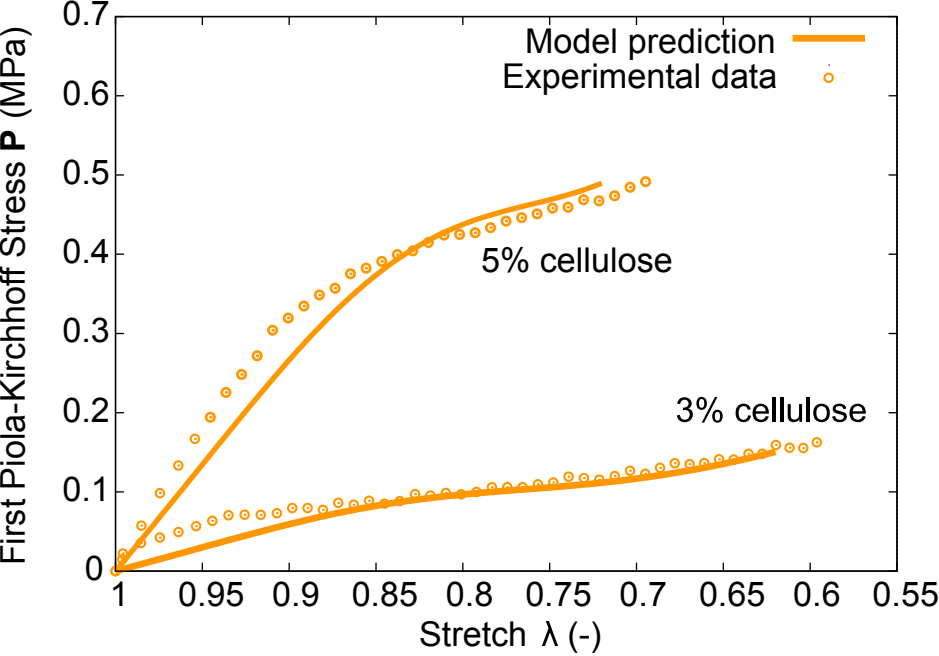

Supplement: Supplementary file 1 [file materials-11-01670-s001.zip › SupplementaryMaterial/Supplementary Material/Figure11-eps-converted-to.pdf]
